# Supplementary figures and images for: Drug screening to identify compounds to act as co-therapies for the treatment of Burkholderia species
Source: PLoS One. 2021 Mar 25;16(3):e0248119. doi: 10.1371/journal.pone.0248119 (PMC7993816; doi:10.1371/journal.pone.0248119)

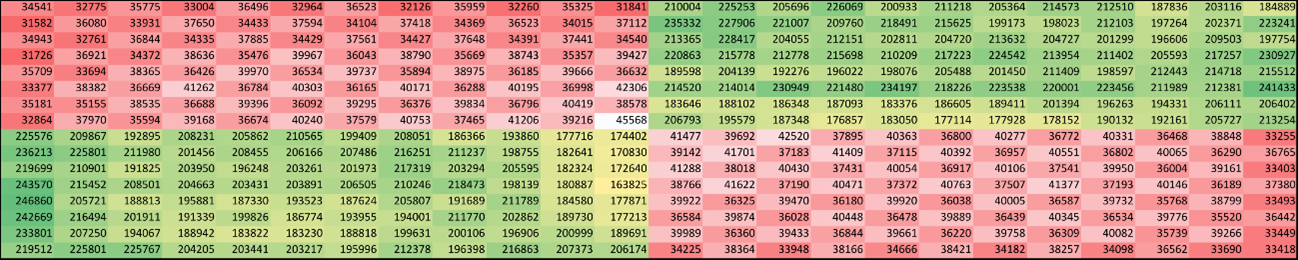

Supplement: S1 Fig — A B. thailandensis culture was harvested and resuspended to a concentration of 8x10^8^ CFU/mL in M9 media supplemented with 730 μM ceftazidime. 45 μl of this suspension (green) and a heat killed control (red) were added to each well in quarters of a 384 well plate. Samples were incubated statically at 28°C. After 20 hours, PrestoBlue was added and the fluorescence read. Intensity of colour indicates the signal strength. Maximum signal variance was 11.2%CV, with Z’ = 0.68 (Calculation: Mean of positive wells = 204,371, SD = 15,179; mean of negative wells = 37,339, SD = 2,532). Relative fluorescence units (RFU) are given for all wells showing significantly decreased fluorescence in edge and corner wells compared to central wells (p = 0.011). Calculation of derived Z (as a worked example of all such calculations in the manuscript): Positive wells: Mean (μp) = 204371, standard deviation (σp) = 15179. Negative wells: Mean (μn) = 37339, standard deviation (σn) = 2532. Difference of means: μp—μn = 204371–37339 = 167032. Sum of standard deviations: σp + σn = 15179 + 2532 = 17711. ZFactor=1−3(σp+σn)|μp−μn| Z = 1 –(3 * 17711/167032) Z = 1–0.318 Z = 0.68. (TIF) [file pone.0248119.s001.tif]

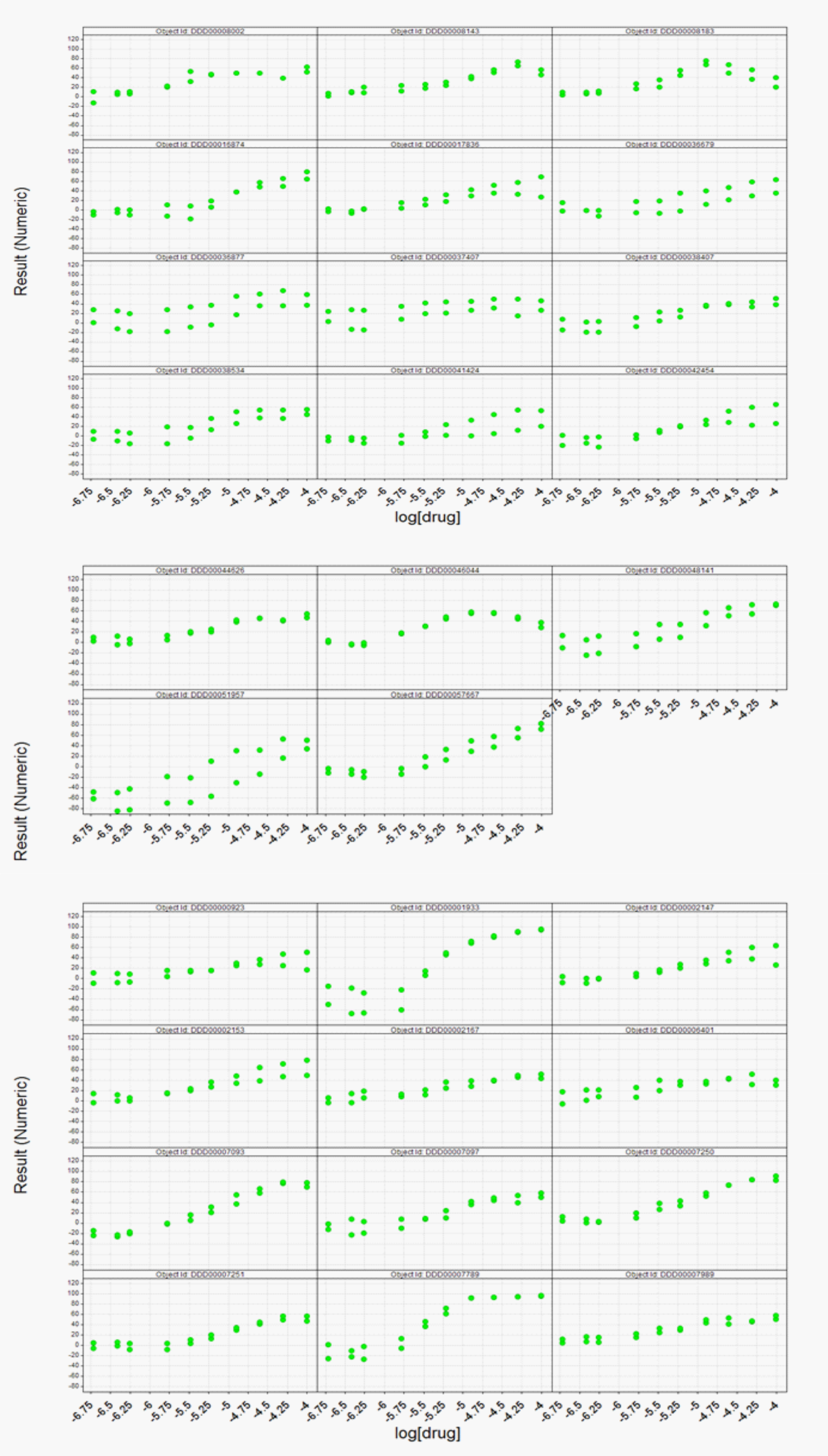

Supplement: S2 Fig — This was added to a 96 well plate containing a concentration response assay performed in duplicate two-fold dilutions of compounds in DMSO. Plates were incubated for 24 hours at 37°C before addition of PrestoBlue and the fluorescence read. The criterion for a positive hit was set as greater than 50% inhibition at the highest concentration tested (100 μM). (TIFF) [file pone.0248119.s002.tiff]

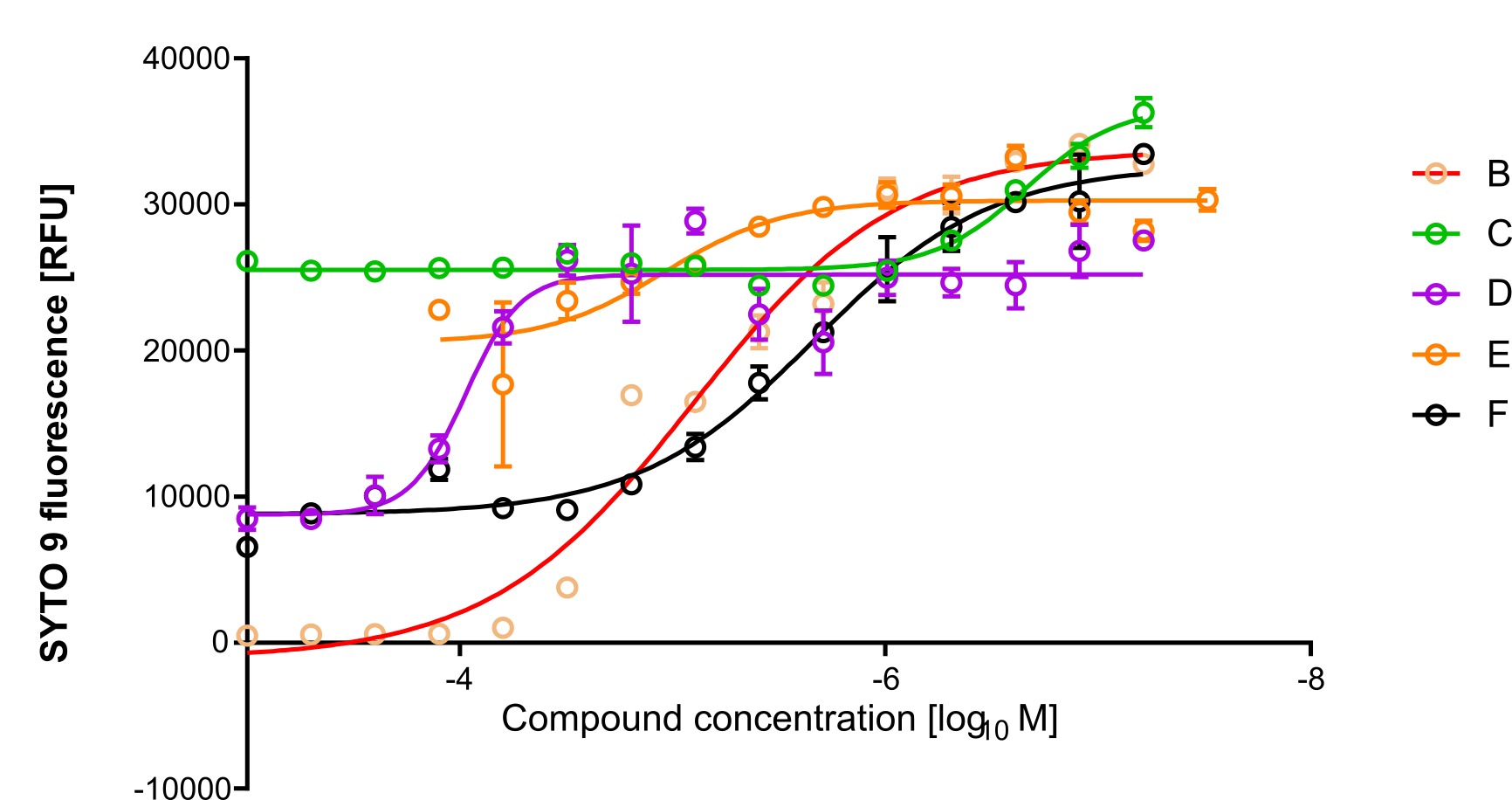

Supplement: S3 Fig — The Live/Dead reagent SYTO9 was used to quantify viability as a function of the membrane integrity of the cell. A B. thailandensis culture was harvested and resuspended to a concentration of 8x10^8^ CFU/mL in M9 media supplemented with 730 μM ceftazidime. This was added to a 96 well plate containing two-fold dilutions of compounds in DMSO. Plates were incubated for 24 hours at 37°C before addition of the Live/Dead cell viability reagents and the fluorescence read. Results show three biological replicates with error bars indicating standard error. The derived IC50 values are shown in S1 Table. (TIFF) [file pone.0248119.s003.tiff]

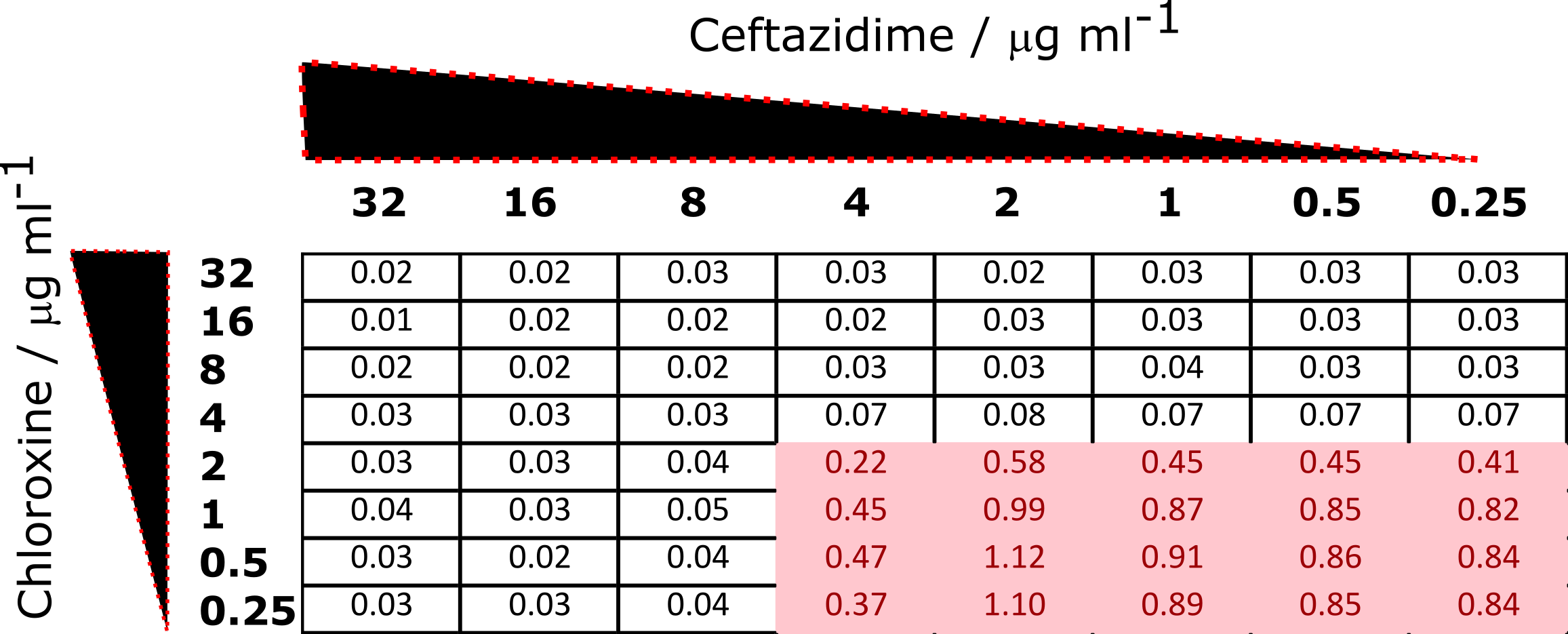

Supplement: S4 Fig — A B. thailandensis culture was diluted to an OD600 of 0.004 in Muller-Hinton broth (MHB; Sigma). Solutions of ceftazidime and chloroxine at 4X final concentration in MHB were prepared by serial dilution from a master stock. Stocks were mixed one part chloroxine stock, one part ceftazidime stock, and two parts B. thailandensis culture (giving an inoculum of ~5 x 10^5^ cfu) in a 96 well plate. Samples were sealed and grown at 37°C statically for 20 hr, following which absorbance at 600 nm was read using a plate reader. Values were corrected for non-inoculated controls. Wells that showed growth (OD600 > 0.1, corresponding with the results of visual inspection; no antibiotic controls showed an OD600 of 0.88 ± 0.1, n = 8) are highlighted in red. The plate reader results were in correspondence with visual inspection. (TIFF) [file pone.0248119.s004.tiff]

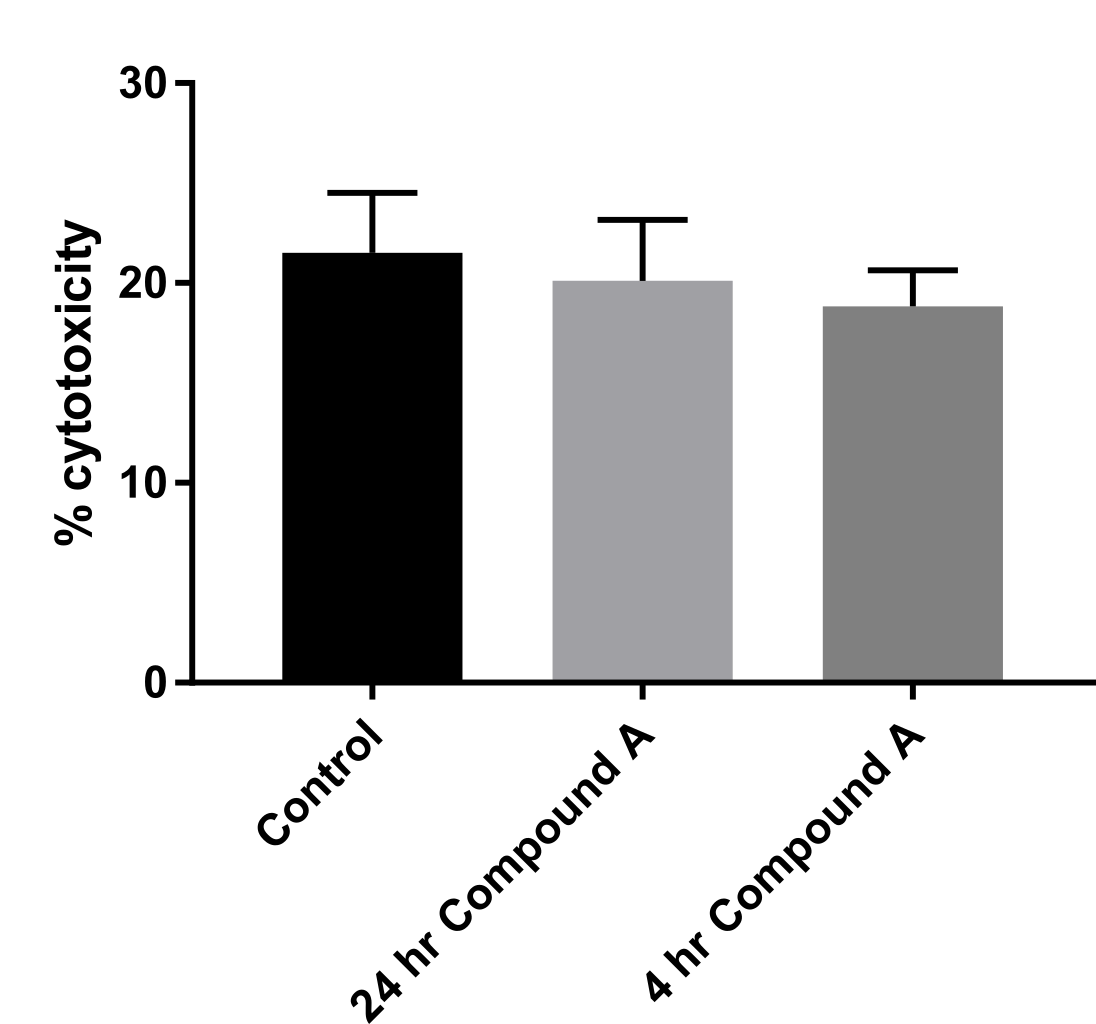

Supplement: S5 Fig — Chloroxine was tested to determine whether it had any cytotoxicity against mammalian cells. Neuroblastoma cells were selected as a representative mammalian cell line that is robust and unaffected by DMSO at concentrations up to 1% (v/v). Cells were plated at 20,000 cells/well in 100 μl Dulbecco’s media. 300 μM chloroxine in 0.5% (v/v) DMSO, or 0.5% (v/v) DMSO (carrier) was added, and the plate incubated for 4 or 24 hours. Cytotoxicity was determined using an LDH cytotoxicity assay kit (Thermo Scientific #88953). Briefly, 10 μl of lysis solution (to indicate 100% lysis) or water (control) was added to untreated wells, and these incubated at 37°C for 45 min. 50 μl of supernatant from each well was added to 50 μl of room temperature assay solution in a 96 well plate (Greiner Bio-One #655201). Samples were incubated at room temperature in the dark for 30 min, and 50 μl of assay stop solution added. Absorbance at 490 nm and 680 nm was read in a M200 Pro plate reader (Tecan), with the difference between these representing LDH activity. % cytotoxicity was determined on a linear scale between the measurements for 100% lysis and water only control. No significant difference was observed between treated and control cells (two-way ANOVA testing for effect of compound or time gives p > 0.5 for each effect). n = 6; image shows means with error bars showing SEM. (TIFF) [file pone.0248119.s005.tiff]
