# Supplementary material for: Drug screening to identify compounds to act as co-therapies for the treatment of Burkholderia species
Source: PLoS One. 2021 Mar 25;16(3):e0248119. doi: 10.1371/journal.pone.0248119 (PMC7993816; doi:10.1371/journal.pone.0248119)
Supplement: S1 Table — The structures, derived IC50 values from the resazurin and SYTO9 based assays and the MIC values are provided for each compound. 95% confidence intervals are shown in parenthesis. IC50 values and confidence intervals were calculated using Graphpad v. 8.3. MIC values were determined as the lowest concentration not showing significant growth. (DOCX) [file pone.0248119.s006.docx]

**S1 Table: Activities of the six most promising compounds.** The structures, derived IC_50_ values from the resazurin and SYTO9 based assays and the MIC values are provided for each compound. 95% confidence intervals are shown in parenthesis. IC_50_ values and confidence intervals were calculated using Graphpad v. 8.3. MIC values were determined as the lowest concentration not showing significant growth.

| Compound | IC_50_ resazurin (μM) | IC_50_ SYTO9 (μM) | MIC (μM) | Compound structure |
| --- | --- | --- | --- | --- |
| **A** | 3.3 (2.8 to 3.8) | N/A^1^ | 30 |  |
| **B** | 24 (21 to 28) | 11 (8.9 to 14) | > 500 |  |
| **C** | 0.067 (0.002 to 0.011) | 0.22 (0.16 to 0.29) | > 500 |  |
| **D** | N/A^2^ | 9.4 (7.1 to 12) | > 500 |  |
| **E** | 6.8 (3.4 to 32) | 11 (4.2 to 28) | > 500 |  |
| **F** | 8.5 (4.8 to 15) | 2.2 (1.5 to 3.0) | > 500 |  |
