# Supplementary material for: Drug screening to identify compounds to act as co-therapies for the treatment of Burkholderia species
Source: PLoS One. 2021 Mar 25;16(3):e0248119. doi: 10.1371/journal.pone.0248119 (PMC7993816; doi:10.1371/journal.pone.0248119)
Supplement: S2 Table — Each compound was tested using the PrestoBlue assay. A B. thailandensis culture was harvested, and resuspended to a concentration of 8x10^8^ CFU/mL in M9 media supplemented with 730 μM ceftazidime. This was added to a 96 well plate containing two-fold dilutions of compounds in DMSO. Plates were incubated for 24 hours at 37°C before the addition of PrestoBlue and the fluorescence read. Results show three biological replicates with error bars indicating standard deviation. All modifications resulted in reduced activity when compared to chloroxine. In cases where the data did not fit to the model used (where no activity is demonstrated at the concentrations used), pIC50 is recorded as N/A. (DOCX) [file pone.0248119.s007.docx]

S2 Table: Hit expansion structures and activity for chloroxine. Each compound was tested using the PrestoBlue assay. A *B. thailandensis* culture was harvested, and resuspended to a concentration of 8x10^8^ CFU/mL in M9 media supplemented with 730 μM ceftazidime. This was added to a 96 well plate containing two-fold dilutions of compounds in DMSO. Plates were incubated for 24 hours at 37 °C before the addition of PrestoBlue and the fluorescence read. Results show three biological replicates with error bars indicating standard deviation. All modifications resulted in reduced activity when compared to chloroxine. In cases where the data did not fit to the model used (where no activity is demonstrated at the concentrations used), *p*IC_50_ is recorded as N/A.

| Structure | Graph | *p*IC_50_ |
| --- | --- | --- |
| A  |  |  |
| A1  | 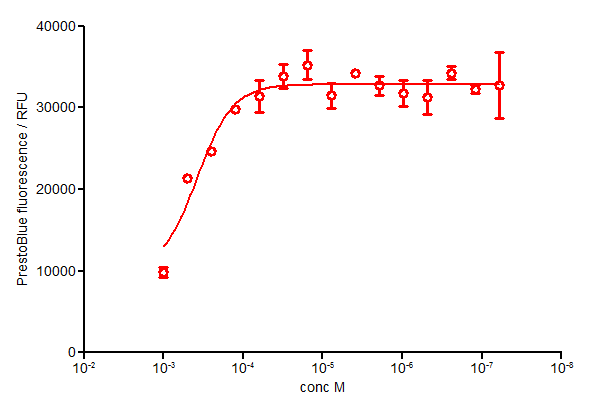 | 3.4 |
| A2  |  | N/A |
| A3  |  | N/A |
| A4   |  | 5.1 |
| A5  |  | N/A |
| A6   |  | N/A |
| A7   |  | N/A |
| A8   |  | 3.4 |
| A9  |  | 3.1 |
| A10   |  | 4.1 |
| A11   |  | 4.2 |
| A12   |  | 5.0 |
