# Supplementary material for: Drug screening to identify compounds to act as co-therapies for the treatment of Burkholderia species
Source: PLoS One. 2021 Mar 25;16(3):e0248119. doi: 10.1371/journal.pone.0248119 (PMC7993816; doi:10.1371/journal.pone.0248119)
Supplement: S1 File — A series of approaches were trialed to identify an effective assay for determining the level of B. thailandensis cells surviving following 24 hours of exposure to 730 μM ceftazidime. The PrestoBlue approach that was eventually selected is described in detail in the main paper. The criteria used for selection was the ability to identify a four-fold difference in initial cell numbers with clear statistical significance; affordability of reagents for over 60,000 test samples; and ease of use in a high throughput setting. (PDF) [file pone.0248119.s008.pdf]

S1 File

Supplementary results

A series of approaches were trialed to identify an effective assay for determining the level of *B. thailandensis* cells surviving following 24 hours of exposure to 730 μM ceftazidime. The PrestoBlue approach that was eventually selected is described in detail in the main paper. The criteria used for selection was the ability to identify a four-fold difference in initial cell numbers with clear statistical significance; affordability of reagents for over 60,000 test samples; and ease of use in a high throughput setting.

ATP measurement

Dilutions of an overnight culture of *B. thailandensis* were tested with the BacTiter-Glo Microbial Cell Viability Assay (Fig 1). Two-fold dilutions in media were taken from a starting cell density of OD_{600} 1.6 (equivalent to approximately 1.6 x 10^9 CFU/ml). For an untreated culture, there is good differentiation between the initial dilutions. However, after six dilutions, the signal reduces to a barely measurable level, where the errors are too high to provide differentiation. As this represents only a 32-64 fold dilution from the initial (high density) culture, this suggests that after treatment with antibiotic, there will be limited signal. Indeed, upon moving to bacterial cultures treated overnight with ceftazidime, it was not possible to reproduce signals detectable above background noise.

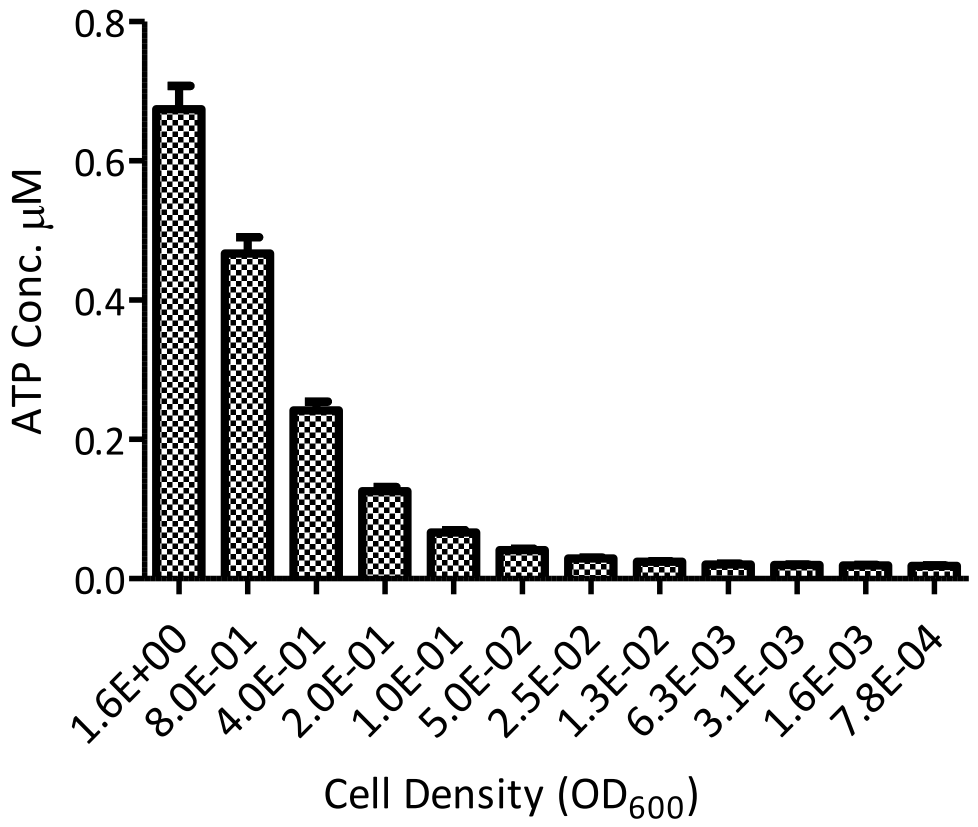

Figure 1: Assay development using Bactiter Glo to measure ATP levels.

ATP levels in an untreated culture of *B. thailandensis* was quantified with Bactiter Glo reagent, in a series of two-fold dilutions with media. Signal from the Bactiter Glo was converted to an ATP concentration using an ATP standard curve in the same media. Results are the mean of three replicates. Error indicates 95% confidence intervals. Z’ for 0.8 to 0.4 = 0.61.

Quantitative PCR measurement of cell numbers

It is possible to quantify cell numbers through real-time PCR. We hypothesized that changes in cell numbers could be accurately determined by comparing the levels of DNA to a standard curve. BTH_II0730, a putative sugar binding protein previously validated for identification of *Burkholderia* species was chosen as the template that would be amplified to determine DNA concentration. This gene is highly conserved in *Burkholderia* sp. The standard curve is linear over five orders of magnitude (Fig 2, upper panel). However, upon testing the assay with serial dilutions of *B. thailandensis*, no discrimination was observed for the reduction in cells number (Fig 2, lower panel). This is hypothesized to be a consequence of background fluorescence caused by LB media. It became evident that the cost and complexity of this method would be prohibitive for use in high-throughput screening. The rate of DNA degradation in dead cells was also a concern as DNA from non-viable cells may still be amplified. A wash step would reduce this risk but also add a further step increasing cost, difficulty, and error. This approach was therefore excluded.

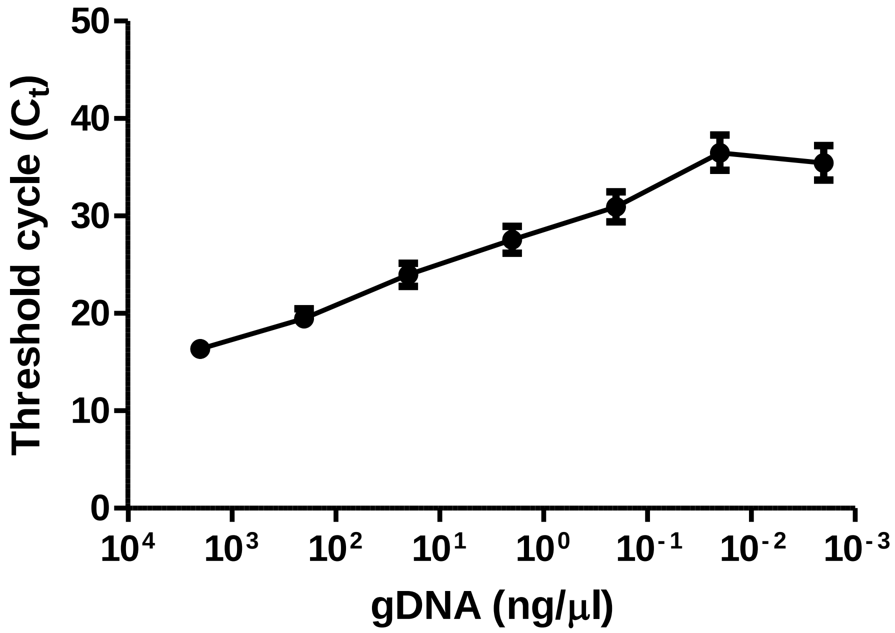

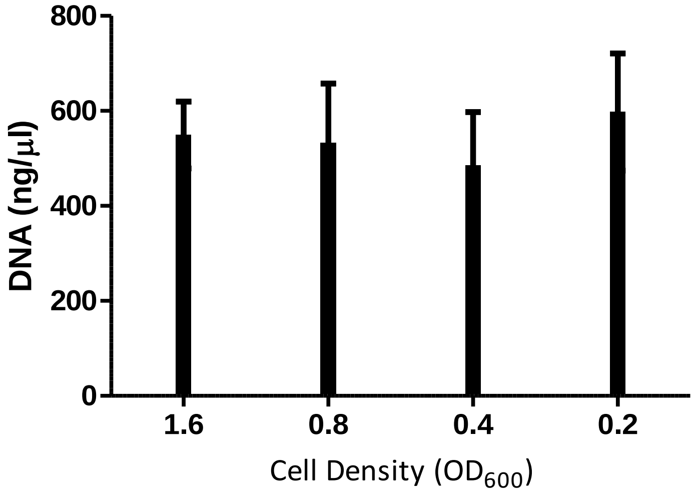

Figure 2: Assay development of cell number determination by qPCR.

Upper: Standard curve created from gDNA dilutions. qPCR of the BTH_II0730 gene from *B. thailandensis* with the SYBR green probe showed a detectable difference in 10-fold dilutions over a good dynamic range from 10^{-2} to 10^{3} ng/mL. A best fit of these data gives $y = -1.5\ln(x) + 26$; $R^2 = 0.99$. Lower: Calculated DNA quantities from a dilution series of *B. thailandensis*. No significant differences are observed between different dilutions.

Plasmid encoded fluorescent protein

This approach used a strain of *B. thailandensis* modified with a plasmid containing red fluorescent protein (RFP) whose expression was driven by the *groS* promoter. The rationale for this approach was that the constitutive expression should give a consistent signal for surviving cells; and the fluorescence should give both sensitivity and a good dynamic range. Despite having a significantly slowed metabolism, antibiotic tolerant cells are still able to produce proteins. Whilst this approach gave excellent resolution in detecting a two-fold difference in seeded cell numbers and displaying a suitable dynamic range (Fig 3), the assay was not suitable for determining the effects of additional compounds on cells. This was due to the expressed fluorophore accumulating in solution and inhibiting the detection of cell number reductions (Fig 4).

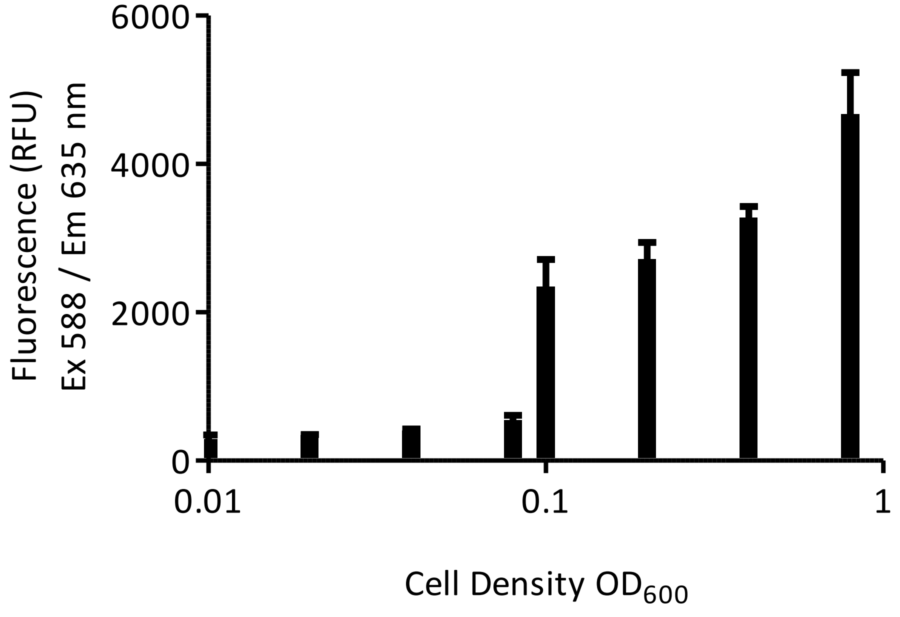

Figure 3: Assay development using recombinantly expressed Red Fluorescent Protein.

Fluorescence of a *B. thailandensis* culture expressing plasmid encoded RFP shows clear resolution of two-fold differences in cell numbers. A culture was grown to OD600 = 1.6 before being harvested and resuspended in fresh LB containing 750 µg/ml chloramphenicol (to retain the RFP plasmid). The culture was adjusted to 8 x 10^8 cfu/mL and two-fold dilutions made in a black-walled 96 well assay plate (Corning, #07-200-567) in LB media. Fluorescence was read at ex 588 nm and em 635 nm using an Infinite M200 Pro (Tecan) plate reader. Z' prime for a two – fold difference from 0.8 to 0.4 = 0.5. Results shown are in triplicate, error indicates standard deviation.

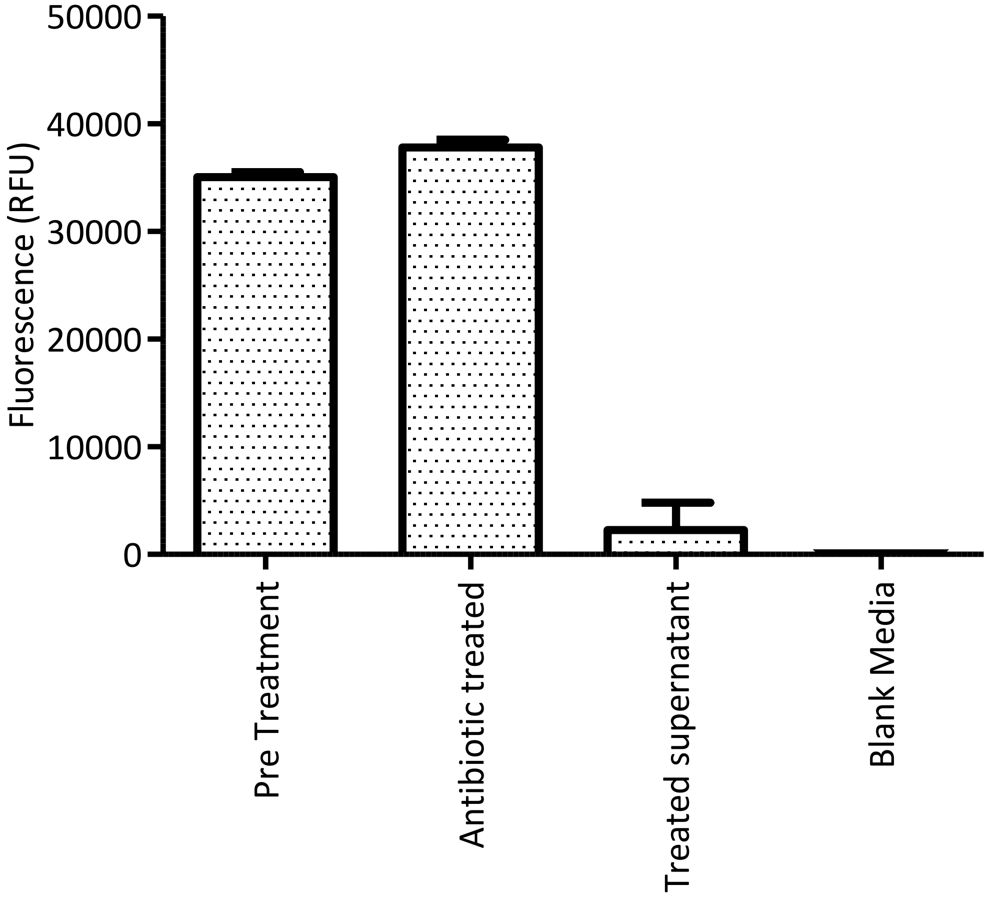

Figure 4: Residual fluorescence of RFP

Fluorescence of a *B. thailandensis* culture expressing a plasmid encoding RFP in the presence and absence of ceftazidime. A culture was grown to OD600 = 1.6 before being harvested and resuspended in fresh LB containing 750 µg/ml chloramphenicol. The culture was adjusted to 8 x 10^8 cfu/mL (pre-treatment) with the same media or media supplemented with ceftazidime to 730 µM (antibiotic treated). Cells were grown for 20 h at 37 °C and fluorescence read at ex 588 nm and em 635 nm using an Infinite M200 Pro (Tecan) plate reader. Antibiotic treated and blank media were similarly measured for comparison.

LIVE/DEAD cell viability staining

LIVE/DEAD® BacLight™ Cell viability staining is a two-color fluorescence assay, combining a membrane soluble green nucleic acid stain (SYTO9), and a red nucleic acid stain (propidium iodide) that does not penetrate membranes. The ratios of dyes used was optimized for *B. thailandensis*. Initially, when LB media was used, this method did not give an acceptable signal to noise ratio (data not shown). However, the use of M9 minimal media reduced background fluorescence. Further optimization included incubation with a breathable membrane. These steps improved the assay to give LIVE / DEAD staining a suitable dynamic range (Figure 5) and differentiating ability. As this stain is more expensive than PrestoBlue, it was not selected for the high throughput screen.

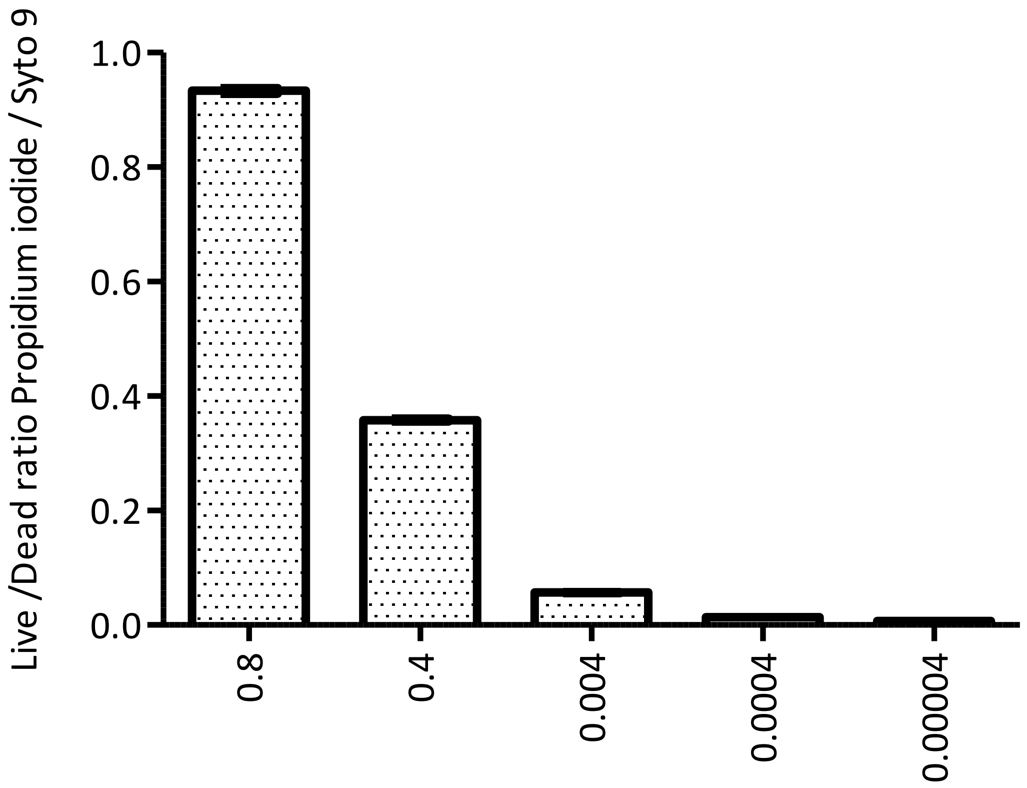

Figure 5: Assay development using LIVE / DEAD cell viability reagent

The LIVE/DEAD reagents SYTO9 and Propidium Iodide were used to quantify viability as a function of the membrane integrity of the cell. A *B. thailandensis* culture was harvested and resuspended to a concentration of $8x10^8$ CFU/mL in M9 media supplemented with 730 μM ceftazidime. Two and ten-fold dilutions of *B. thailandensis* were added to a 96 well plate. Plates were incubated for 24 hours at 37 °C before addition of Live / Dead cell viability reagents and the fluorescence read. Results show four biological replicates with error bars indicating standard deviation.

Experimental Procedures

ATP measurement

A culture of *B. thailandensis* was grown to a cell density of OD_{600} 1.6, equivalent to 1.6 $x10^9$ CFU/ml. Cells were harvested and resuspended in an equal amount of LB supplemented with 730 μM of ceftazidime. Samples were incubated statically for 24 hours at 37 °C. ATP concentration was determined against an ATP ladder produced from a 10 µM stock solution of ATP in LB which was serially diluted in 10-fold dilutions in a 96 well plate. Aliquots of 100 µl for all samples; t0, t24 and ATP standards were added to wells of an opaque, white walled 96 well plate (Corning, #3917) and 100 µl BacTiter-Glo reagent (Promega, #G8230) added. Plates were mixed using an orbital shaker and incubated at room temperature for 5 minutes before luminescence was read using an Infinite M200 Pro (Tecan) plate reader.

qPCR evaluation of cell numbers

The experiment was designed according to the minimum information for publication of quantitative real-time PCR experiments (MIQE) guidelines [4]. Primers were designed for BTH_II0730 using Applied Biosystems Primer Express 3.0 software for a 300-400 bp amplicon. Genomic DNA (gDNA) was extracted from a stationary culture of *B. thailandensis* using a GeneJET Genomic DNA Purification Kit (ThermoFisher, #K0491) and quantified using a NanoDrop 2000c spectrophotometer. gDNA was diluted to 2 ng/µl and 10-fold dilutions were made in distilled water for a standard curve. Two-fold dilutions of *B. thailandensis* from a stationary culture in LB were produced from OD_{600} 1.6 to 0.2. qPCR was carried out using SYBR Green Real-Time PCR Master Mix (ThermoFisher) on a Step One Real-Time PCR System (Applied Biosystems).

Plasmid encoded fluorescent protein

The plasmid pBHR4-groS-RFP [5] was conjugated into *B. thailandensis* strain E264. A culture was grown to OD_{600} 1.6 before being harvested and resuspended in fresh LB containing 750 µg/ml of chloramphenicol. The culture was adjusted to 8 x 10^8 cfu/mL and two-fold dilutions made in a black-walled 96 well assay plate (Corning, #07-200-567) in LB media. To test with antibiotic, cells were harvested by centrifugation and resuspended in LB media supplemented with 730 µM of ceftazidime. Fluorescence was read at ex 588 nm and em 635 nm using an Infinite M200 Pro (Tecan) plate reader.

LIVE/DEAD cell viability staining

The LIVE/DEAD *Bac*Light bacterial cell viability kit (Invitrogen, #L7012) was used for this assay. A culture was grown to OD_{600} 1.6 before being harvested and resuspended in fresh LB or M9 media containing 730 µM of ceftazidime. Two and ten-fold dilutions were made with the same media, and 100 μL added to a black walled 96-well plate (Corning, #3904), and the samples incubated statically for 24 hours at 37 °C. A master mix of equal volume SYTO9 to propidium iodide was prepared and 3 µl added

to wells and mixed thoroughly. Plates were incubated at room temperature in the dark for 15 minutes and fluorescence was read at ex 480 / em 500 nm for SYTO9 stain and ex 490 / em 635 nm for propidium iodide.

Cytotoxicity assay

The cytotoxicity assay was performed using the SH-SY5Y human neuroblastoma cell line. 10,000 cells/well were seeded into a 96 well tissue culture plate and grown overnight in 100 μL DMEM supplemented with fetal calf serum at 37 °C in 5% CO_2. 10 μL of 30 μM chloroxine in culture medium with 0.1% DMSO was added to test wells and incubated for 4 or 24 h as above. Cytotoxicity was determined using an LDH cytotoxicity assay kit (Thermo, #88953), following the manufacturer's instructions. 100% cytotoxicity was determined by adding 10 μL of the kit cell lysis reagent to control cells and incubating at 37 °C for one hour. 0% cytotoxicity was determined using culture medium. 10 μL of water was added to the control cells one hour before readings were taken to provide identical volume to test samples. Statistics were performed using *R*.
