# Supplementary material for: Drug screening to identify compounds to act as co-therapies for the treatment of Burkholderia species
Source: PLoS One. 2021 Mar 25;16(3):e0248119. doi: 10.1371/journal.pone.0248119 (PMC7993816; doi:10.1371/journal.pone.0248119)
Supplement: Attachment — Submitted filename: Response to reviewers.pdf [file pone.0248119.s010.pdf]

Response to reviewers:

We thank the reviewers for their careful reading of the manuscript, and for the additional suggestions for further improvement. We have revised the manuscript to meet all of these comments.

We have also revised the manuscript as requested by PLoS One to meet the PLoS style.

Reviewer #1: I am Reviewer #2 from the prior submission. I have read and appreciate the responses made by the authors to my comments and concerns. I have also read the insightful comments and concerns of Reviewer #1 from the prior submission and I am generally satisfied that the authors have addressed those points. The thoughtful discussion contributions made by both parties are very much appreciated.

It is my view that the text of the paper alone reads well at present. It is quite straightforward and I believe that the current version is better than the last.

We thank the reviewer for these comments. It was our hope that the text would read well.

However, I am frustrated about the way the Figures and Tables (including those that are Supplementary) are integrated with one another and the text. In order to follow along, one must open the Results section of the text and open the corresponding (in-text) Figures/Tables and also have the Supplementary Figures/Tables open as well as the Experimental Procedures section of the text to gather all of the information needed to understand a given result. This frustration has been mentioned by other, prior Reviewers (see discussions pertinent to readability and sequence of data presentation). This frustration seems to extend beyond the routine and expected awkwardness of navigating a manuscript before it has been accepted and transformed into the final publication format. It makes me wonder if a full re-working of the manuscript is necessary rather than merely updating it piecemeal to satisfy the comments garnered in each additional round of review. No doubt the document has been reviewed and updated many times.

A key concern I have is that the authors seem to refer to elements of figures (particularly Supplementary Figures), which I cannot find. This makes me wonder if I am looking at the same version of the Supplementary Figures as the authors are looking at. Please see below for line-by-line examples.

I thus conclude that from a scientific standpoint, I am satisfied that my concerns have been addressed. However, I'd encourage the authors to sit with the paper and take a very careful look at all of the references to in-text / Supplementary Figures and Tables to make sure that everything is coherent.

We appreciate the reviewer's comment about careful checking of the figures and tables, especially in the light of the well-made line by line comments. We have given the manuscript thorough checking to make sure that each reference to figures, tables and supplementary material is correct.

Line-by-line Comments

Lines 95-99: "Previous studies have shown that approximately 0.1% of B. thailandensis cells survive for 24 hours following treatment with 100X MIC." Likewise, can you quote a survival percentage for B.P. from the literature – if available?

These data are available in the literature, and we have added this to this section (*revision – line 101)*.

Line 166-169: "pIC50, hillslope and maximal effect were used to further down select to six compounds (A-F), all of which displayed a pIC50 > 5 (Figure 3A, Table 1, Supplementary Figure 8, Supplemental Table 1)". Firstly, it is tremendously confusing to make a statement and then provide substantiation of the statement by referring to an in-text figure, an in-text table, a supplementary figure and a supplementary table. It is almost enough to cause the reader to give up on trying to independently review points of evidence for the claim. There must be a better way to convey which parts of the claim are substantiated by evidence in which in-text figures and tables and in which Supplementary figures and tables. Secondly, I don't see the structures for Compounds A-F in Supplementary Table 1. Are they supposed to be here or elsewhere – for example in one of the other tables or figures? Moreover, which letter (A-F) is chloroxine in each figure/table?

We appreciate the reviewer's point about the challenge of viewing these data. To address this, we have broken these data into two, highlighting the primary assay for Fig 3 and Table 1, and the secondary assay in the Supplementary figure/table. [Revision – line 293.]

We have added the structures of compounds A-F to Table S1 as the reviewer suggested.

Chloroxine is compound A – we have clarified this in the text at the point that the reviewer noted, which is a good idea. [Revision – line 294-5.]

Line 179-180: The text suggests that there is a depiction of chloroxine's antimicrobial activity with an MIC of 4 ug/mL in Supplementary Table 2. I do not see any information regarding this in Supplementary Table 2. Yet again, there is discussion of Reviewer #1's comments included in the information for the authors from the 2nd submission of this manuscript to PLOS ONE. Here, it sounds like the authors are directing Reviewer #1 to see Supplemental Table 1. I don't see this information there either. Please address.

We apologise for the lack of clarity here. The CLSI method calls for visual inspection of wells, which does not lend itself well to representation except as a determined MIC. The determined values are listed in Table S1. We have removed the reference to table S2 which was in error. The results shown in Fig S4 are consistent with the determined MIC, providing a further validation of the MIC result.

Figure 1: Please state what the positive control is in the figure legend. Otherwise one must dig and look in the Experimental Procedures section to find it.
This is an excellent suggestion and we have added this to the figure legend. [Revision: lines 251-2.]

Response to Reviewer#2 Comment from Prior Submission: Previously, I requested an example of how the 'Z' factor is calculated. In your response, you indicated that you have included one and I was eager to take a look at that. However, I cannot find an example in the legend of Supplementary Figure 1, which is where I was directed to look. This makes me question if you have perhaps submitted the wrong version of the Supplementary Figures and that I am consequently now looking at the wrong version (?). Perhaps I am not looking at the final draft of your updates to the Supplementary Figures (?). This could account for my ongoing confusion. For context, this is what I see:

Supplementary Figure 1: Assay development using Bactiter Glo to measure ATP levels.
ATP levels in an untreated culture of B. thailandensis was quantified with Bactiter Glo reagent, in a

series of two-fold dilutions with media. Signal from the Bactiter Glo was converted to an ATP concentration using an ATP standard curve in the same media. Results are the mean of three replicates. Error indicates 95% confidence intervals. Z' for 0.8 to 0.4 = 0.61.

Thank you for your careful consideration to these matters.

We apologise here, as we unfortunately misunderstood the reviewer's previous request. We have included a sample calculation in Fig S1, which is the ideal place for a detailed example.

Reviewer #2: This reviewer acknowledges that the author and colleagues have addressed all the comments and concerns posed in the review, included pertinent data, added missing information and corrected typographical errors.

We thank the reviewer for their comments.
