# Supplementary material for: Drug screening to identify compounds to act as co-therapies for the treatment of Burkholderia species
Source: PLoS One. 2021 Mar 25;16(3):e0248119. doi: 10.1371/journal.pone.0248119 (PMC7993816; doi:10.1371/journal.pone.0248119)
Supplement: Attachment — Submitted filename: Barker et al Response to reviewers 0920.docx [file pone.0248119.s009.docx]

Barker et al. Response to reviewers

We are grateful to the reviewers for their thorough and clearly communicated thoughts on our manuscript. We have responded to these thoughts in detail below, and altered the manuscript to reflect the reviewers’ comments. We agree that the manuscript is substantially improved thanks to these changes and thank the reviewers again for their thoughtful suggestions for improvement.

*Reviewer #1: In this manuscript the authors attempt to identify co-therapies for Burkholderia along with the current ceftazidime treatment. The authors suggest that from their screen of 61,250 compounds, the compound chloroxine has potential to be used as a co-therapy for B. pseudomallei. Several things including poor readability, non-sequential flow, lack of consistency, overstating importance of the work have reduced enthusiasm for this manuscript. Despite addressing most comments from the previous reviewers, this manuscript still has several shortcomings as mentioned below:
1. In the title, the authors mention ‘co-therapies for treatment of pathogenic Burkholderia’. However, all the studies performed except figure 8 utilizes B. thailandensis, the avirulent form. The title seems like an overstatement.*

We have removed the word “pathogenic” from the title.

*2. Line 70 – ‘neutrophils being particularly preferred in mice’ – Reference 15 states that neutrophils in BALB/c mice are important in fighting Bp infection. Bp does not prefer to invade neutrophils in all mice. This should be corrected.*
We have removed this part of the sentence.

*3. This manuscript displays lack of rigor in writing the manuscript sequentially to create a logical flow. For example: Figure 1 showing structure of the compounds comes before figure 2 which describes ceftazidime dose response using PrestoBlue assay and before any figures even describing the 2 finally chosen compounds.*
We appreciate the referee’s point of view. We have revised the manuscript to improve the flow and have removed redundant material to make it more readable. In particular, we have moved Figure 1 as the referee requested.

*4. Figure 2:
a. It is unclear how the initial CFU was determined.
b. Since viability analysis using Presto blue assay is performed after 20 hours of incubation, it might be a better idea to compare the actual CFUs at that time with the RFU readings. This will provide better correlation of the bacterial numbers to RFU. This experiment is essential since the authors are using 8x10^8 bacteria for rest of the experiments based on figure 2 data.
c. The authors have used 0.1M NaOH to dilute 10mg/ml of ceftazidime. Depending on the dilution process, would the amount of NaOH used change the alkalinity of the bacterial suspension after adding ceftazidime? This is particularly important to know since there is no vehicle control performed for ceftazidime.*

a. This is a very good point. We have added the method of calculation into the methods section (bacterial strain and culture conditions).

b. We respectfully disagree with the referee’s suggestion. In our preliminary investigations, we reviewed CFU enumeration. We found that this was not reproducible and was poorly correlated with the outcome of other detection methods (e.g. those outlined in the supplementary material). The PrestoBlue method (and most of the other methods tested) were better correlated, in contrast. We suspect that relying on viable CFU enumeration would miss a substantial proportion of cells that have low metabolic activity in *Burkholderia*. Since our experiments were aimed at measuring these cells in particular, this would rather invalidate the study.

c. This is a fair point, as the carrier may affect the pH of the media. We note that this method has been used extensively by others (e.g. DOI: 10.1128/AAC.02793-14 which is cited by many similar studies). A test of the media showed that the effect of the carrier is to raise the pH from 7.1 to 7.4 (M9 media is well buffered so the effect is comparatively small). The latter pH is closer to the optimum growth pH for *Burkholderia* in soil (doi: 10.1007/BF02930965). In the context of this experiment, the relevant comparisons are between the samples rather than a carrier control. We note that the sample seeded with the highest concentration of cells showed nearly as many surviving cells as the untreated sample (as expected for *Burkholderia* in these conditions), so the effect of pH was clearly not limiting to cell growth.

*5. Figure 3: How did the authors decide on 30uM compound concentration? The initial bacterial CFU used in this experiment is not mentioned. Was it still 8x10^8?*

Yes, the bacterial cfu added was the same. We have clarified this in the methods section.

*6. Figure 4: The authors mention compounds A-F on line 176-177 but show data for A and B only.*

We have included data for compounds **C-F** in this figure. More data for compounds **C-F** is provided in supplementary information as they are not the main focus of the manuscript.

*7. Figure 5:
a. This figure shows the IC50 determination for compound B using Live/Dead cell viability assay. Since the authors move on with compound A, why is the same assay not done with compound A? The reason for lack of this data is unclear.
b. For live/dead cell assay used here, were the starting bacterial numbers optimized? This assays allows for 2x10^8 E.coli and 2x10^7 S. aureus, both smaller in size than Burkholderia. Since 8x10^8 cells were used here, is it possible that the Live/dead ratio observed might not be optimal. This raises questions regarding usefulness of this data.*

We have moved this figure to Supplementary information (this now includes the data for compounds **B-F**). Compound **A** (chloroxine) was not studied in this assay as it absorbs at one of the relevant wavelengths (noted in the supplementary information).

The starting bacterial numbers were optimised. These data were already in the supplementary information. As the ceftazidime kills approximately 90% of the bacteria rapidly, the assay is detecting at most 8x10^7^ cells (and usually fewer), so is in the expected range for Gram-negative bacteria.

*8. Figure 6:
a. This figure is not easy to read. Please increase the symbol size to make it obvious. The X-axis scale should appropriately reflect the time points.
b. How was the dosage of chloroxine decide at 30uM when the does response for compound A was not performed as in Figure 5 for compound B?*

a. We have amended the figure to increase the symbol size and make the time points clearer.

b. The dosage of 30 μM was determined from the dose response curve in Figure 4, where 30 μM is the lowest concentration of chloroxine that reduced the signal to baseline. As described above, chloroxine gave a compound effect with the Live/Dead assay so no effect could be measured here.

*9. Figure 7:
Since the activity of these compounds are compared with the chloroxine, the same experiment should demonstrate chloroxine activity for comparison. Also, the authors do not report the compound concentrations used here.*

This is a good point that requires clarification. The equivalent data for chloroxine are presented in Figure 4, in experiments that were highly reproducible. We have clarified in the figure legend that the relevant data are already in the paper to avoid duplication.

*10. Figure 8:
Similar to previous reviewer #2, I wonder why there is a difference in the basal level between Bp and Bt. Although the authors mention difference in assay values, the authors do not mention what amount of Bp they started with which could be the confounding factor for this difference.*

The reviewer raises a very fair point. The inoculums of each bacterium used were the same. We have amended the figure legend to reflect this. Co-author Gray’s experience of a range of other organisms is that differences in the basal level are common and cannot always be explained.

The bacteria were also cultured in slightly different conditions (as Bp was handled at Containment Level 3), which may have a minor effect on the fluorescence detected.

*11. Supplementary figures are not referenced wherever required, especially the ones showing all the other assays performed before deciding on prestoblue assay.*

This is a fair point. We have added additional references to the supplementary material in the results and discussion sections.

*12. Supplementary Figure 4:
a. Although it is important to show the cytotoxicity of chloroxine on mammalian cells since Bp is an intracellular pathogen, the authors provide no rationale for using neuroblastoma cells. Bp is a lung pathogen so using alveolar macrophages, lung epithelial cells or any other immune cells would be more in line with the infection.
b. Why was 300uM chloroxine used for this assay?
c. It would help the manuscript significantly if the authors measure actual intracellular Bt and/or killing in relevant cells like macrophages. Performing cytotoxicity of chloroxine in irrelevant cells does not add anything to the manuscript.*

a. We chose neuroblastoma cells as they grow readily and are robust to manipulation. They are not affected by 1% DMSO (the carrier for chloroxine in this experiment. The PI’s institute has access to a good range of cell lines and after discussion we felt that this was the most appropriate to meet the aims of the experiment.

We would note that Bp is not exclusively a lung pathogen: it is a pathogen that has multiple routes of infection which can disseminate rapidly and affect many tissues. In addition, this is a test of **cytotoxicity**. If a compound is going to be used clinically, it cannot show toxicity against *any* cells.

b. Our assays indicated an IC50 of 2 μM; we tested for synergy with ceftazidime at reducing surviving cells at 30 μM to easily exceed this (Figure 6). We tested for cytotoxicity at ten times the latter concentration to demonstrate that there would be no cytotoxic effects at well above the effective dose. Antibiotics are generally administered at well above the therapeutic dose to account for the reduction in concentration between doses. This experiment was designed to demonstrate that this would be possible with this compound.

c. We disagree strongly that this experiment is irrelevant, for the reasons given above.

*13. The authors fail to discuss several limitations of their work in the discussion section.
a. Since Bp is an intracellular pathogen, it is important to know if chloroxine is a good therapy for killing intracellular bacteria. The authors should at least discuss this in the shortcomings of their studies or as future direction in the discussion.*
*b. Line 301-302 – ‘identified chloroxine as a potential co-therapy to treat with B. pseudomallei.’ Several additional experiments are required to make this statement which the authors should discuss.*

The referee makes fair points here. We have added further comments to the discussion to address these points.

*14. There are several inconsistencies in the paper:
a. The media used appears to be different – M9, LB or Muller-Hinton Broth (Supplementary fig 3). Could the authors please explain this inconsistency.
b. Line 206 mentions use of 50uM chloroxine whereas the corresponding figure mentions 30uM. What is the actual concentration used?*

a.

LB is the most suitable media for growing *B. thailandensis* for experimental work. This is consistent with previous work and allows comparison with previous results of our institution and others.

However, LB interferes with many of the detection modes that we tested (including resazurin dyes, LIVE/DEAD assays and ATP detection). We therefore used M9 media to allow for use of these detection modes. As all of these experiments are carried out in the presence of 400 ug/mL ceftazidime, surviving cells will be in a low metabolic state so there is little difference between rich and minimal media.

As was explained in the experimental section, the MIC experiment was performed using the CLSI recommended protocol for consistency with the rest of the literature. This protocol requires the use of Muller-Hinton broth.

b. We thank the referee for pointing out this typographical error – 30 μM is the correct value and we have amended the manuscript.

*15. Line 192-193 – ‘Chloroxine demonstrated antimicrobial activity with MIC of 4ug…’ – Which figure shows this data?*

These data are now recorded in Supplementary Table 1. The CLSI protocol requires determining the MIC by eye, and it is rather difficult to present such an image. This result is also consistent with the much more informative data shown in Supplementary Figure 3 (now 9).
 *16. Line 301 – chloroxine as a potential ‘a’ co-therapy – extra ‘a’*
 We thank the referee for noting this typographical error, which has been removed. *17. Line 309 – chloroxine, has known bacteriostatic – ‘C’ should be capital.*
We disagree that chloroxine should be capitalised. Chloroxine is the common name of the chemical (which dates from 1888, when chemicals still got individual names)! The trade name, which would be capitalised, is Capitrol.

*18. Line 331-332 – based on the description in the paragraph, the conclusion ‘ The hit expansion validates chloroxine as a co-therapy for ceftazidime’, sounds like a stretch.*

Yes, this is a fair point. We have re-worded this sentence to make the point that the effect of chloroxine is specific, and that it potentiates the effect of the ceftazidime.

*19. At several occasions, the authors mention that the study has demonstrated a phenotypic assay which can identify compounds that act as co-therapies. However, it is unclear if the authors have developed a novel assay since the assay is use of a commercially available dye, Presto Blue.*

We have demonstrated that the configuration of our assay is effective for identifying compounds that reduce the number of surviving *Burkholderia* cells when treated with the current frontline therapy, ceftazidime. This required a substantial amount of development, both to identify the best reagents, and to optimise the conditions to provide a robust assay suitable for HTS. This development of the assay to use and conditions to use it in for this species and this purpose is the novelty of our work.

*20. The authors should improve figure legends so that they have clear and all important and relevant information*

We have amended the figure legends as requested by the referees.

*Reviewer #2: The authors describe the establishment of a phenotypic high-throughput-assay, which entails the cell viability reagent PrestoBlue™. The assay is subsequently employed to screen a library of 61,250 compounds, identifying one, chloroxine, which, based on included in vitro data, represents a putative co-therapy for administration with ceftazidime in the treatment of Burkholderia pseudomallei infection. B. pseudomallei is a highly pathogenic gram-negative bacterium, which is a biothreat agent. It is found in water and soil in many parts of the world, including those regions which are home to large, resource-poor human populations. B. pseudomallei infection is both difficult to treat and costly as well with guidelines recommending that (eradication) therapy occur over a minimum duration of 3 months. Thus, any agent that improves upon the efficacy of the standard antimicrobial regimens for B. pseudomallei infection is of great significance to the discipline.

This reviewer acknowledges that the author and his colleagues invested a great deal of time and effort into re-writing the manuscript and adding pertinent data to appease the comments and criticisms of those who reviewed the initial manuscript submission. While this reviewer did not read the initial submission, the Reviewer Comments and Author Responses were read. When these are considered in light of the current version of the manuscript, it is evident that the process, while laborious, has strengthened the work as a whole.*

*To briefly address Key Points that PLOS ONE instructs reviewers to consider: 1) The author’s claims do appear to be placed in the context of the previous literature, which itself is treated fairly - contextualization appears to have been particularly strengthened by guidance from those who reviewed the initial submission 2) The data and analyses do appear to fully support the author’s claims and additional evidence is not obviously required 3) The protocols, algorithms and methods appear to be provided in sufficient detail to enable use by the reader including potential replication *except where indicated below* 4) The manuscript is well organized and is clearly enough written to be accessible to non-specialists, including this reviewer *except where indicated below**

S*pecific Line-by-Line Comments:

Line 20-21, Line 78-79: “[B. pseudomallei] is naturally resistant to most currently available antibiotics.” This is an overstatement, which should be altered to reflect the fact that, while some antibiotics are not appropriate for therapy of B. pseudomallei infection (e.g. penicillin, ampicillin, first-generation and second generation cephalosporins, gentamicin, tobramycin and streptomycin), multiple currently available antibiotics are effective alone or in combination with one another (ceftazidime, imipenem, meropenem, TMP-SMX, doxycycline, amoxicillin-clavulanate). References: The Sandford Guide to Antimicrobial Therapy 2018 by Gilbert et al. and Mandell, Douglas, and Bennett’s Principles and Practice of Infectious Diseases 9th Edition by Bennett et al.*

We agree with the referee’s point and have re-worded this to say that it has resistance to *many* antibiotics, rather than most.

*Line 21-22, Line 59, Line 81: “The most efficacious treatment for melioidosis requires at least two weeks of intravenous treatment with ceftazidime or meropenem”. Please include imipenem which many guidelines cite as equivalent to ceftazidime and meropenem in this application. References: Gilbert et al. and Bennett et al. (above)*

This is a very good point and we have amended the text as suggested. We have added the references suggested to the text also.

*Line 27: Please provide an example of how the Z’ factor was calculated. While this reviewer acknowledges that the formula used has been included, as a non-specialist, an example of the calculation that was done and which parameters were used would be informative.*

This is a very good idea to assist the reader. We have provided the calculation in the legend of supplementary figure 1.

*Line 126-127: It is not entirely clear to this reviewer why a 400 microgram per milliliter, 100x MIC dose of ceftazidime is necessary for this application, though this parameter may be a byproduct of the prior version of the study that was more focused on bacterial subpopulations highly resistant to antibiotics. Please include a more complete explanation of why such a high dose is needed. Note that, according to CLSI M45 ED3 (2016) Table 21, the ceftazidime breakpoints for B. pseudomallei in micrograms per milliliter are S: /=32. Also, are the B. pseudomallei (i.e. B. thailandensis) being regarded as “antibiotic tolerant cell populations”? If so, explain why. If not, remove this statement, which may be a hold-over from a past version of the document.*

These experiments were designed to identify compounds that have activity against the sub-population of cells that show tolerance to peak ceftazidime concentrations. 400 mg/mL is approximately the peak blood concentration of ceftazidime given to an adult with septicaemia (according to NICE guidelines, 2020; 2 g dose given IV, an average adult has between 4 and 6 litres of blood depending on weight and sex). We have noted this briefly in the text. Similar studies have used similar concentrations.

Recent papers have reported MIC values of between 2 and 6 μg/mL for ceftazidime against *B. pseudomallei* (Khosvari et al. 2014; Dutta et al. 2017; Ross et al. 2018, cited in the main paper). We determined an MIC for *B. thailandensis* of 4 μg/mL (Supplementary Figure 9). Our levels used are consistent with the recent literature (and our own experiments).

*Line 142-143: “The assay was then optimized for use in 384 well plates with automated dispensing of reagents.” Please explain briefly what was entailed in the optimization – did this involve actions to mitigate the edge effects noted in Supplementary Figure 1? To this end, in Supplementary Figure 1, it is difficult to discern color differences between the wells though the figure legend appears to indicate that this should be noted. Please address this.*

We have amended line 142 to include the key parameters that were amended (reaction volume; adding a seal to minimise evaporation; optimising the temperature to provide optimal reproducibility and minimise evaporation), in addition to using better liquid handling as was already noted. The details were available in methods but we agree that explicitly noting these will be helpful for readers. These were largely necessary to mitigate the edge effects, but also more generally reduced the variability between wells.

We have amended the spectrum gradients in Supplementary Figure 1 to make the differences between wells more striking.

*Line 166-170: “…criteria for a positive hit was set as greater than 50% inhibition at the highest concentration tested (100 micromolar)”. At some point in the text, please provide the definition of IC50 and provide the definition of MIC highlighting for non-specialist readers that these concepts differ. Also, a number of different representations for drug or compound concentration are used throughout the manuscript (micromolar, microgram per milliliter, pIC50). Though it may not be possible in all circumstances (such as if it detracts from emphasis on the meaning and trends pertaining to pIC50), as much as possible, please try to convert all concentrations to the same units. Also, at some point in the text, explain whether or not the concentrations of ceftazidime and chloroxine used with antimicrobial effect in this study would be physiologically achievable in a human patient. One reference which provides an estimate of peak ceftazidime concentration in serum (70 micrograms per milliliter, when administered intravenously) is Manual of Clinical Microbiology 12th Edition Volume 1 Page 1229 by Carroll et al.*

This is a good point. For consistency, we have converted all concentrations to molarities. We have provided the equivalent concentration in micrograms per millilitre at the first mention of ceftazidime to allow for ready comparison with the literature. We have added the equation used to calculate IC_50_ to the experimental section and have defined this on first use in the main text.

*Line 177-180 / Figure 5: Regarding the SYTO9 LIVE/DEAD assay, in the Supplementary Data at Line 161-162 the statement is made “An additional concern with the LIVE/DEAD reagent is that the wavelengths used are known to be troublesome for compound effects”. Please either remove this statement from the Supplementary Data section (the indication that it is more expensive than PrestoBlue is an adequate explanation of why it was not selected at that point in the text) or explain why wavelengths were not troublesome for the data presented in Figure 5.*

We have removed the statement from the supplementary data section as the referee correctly notes that it is superfluous.
